# Sorafenib and DE605, a novel c-Met inhibitor, synergistically suppress hepatocellular carcinoma

**DOI:** 10.18632/oncotarget.3656

**Published:** 2015-03-26

**Authors:** Xiufeng Jiang, Kang Feng, Ye Zhang, Zengyao Li, Fan Zhou, Huiqiang Dou, Tong Wang

**Affiliations:** ^1^ Department of General Surgery, Wuxi People's Hospital, Wuxi, China; ^2^ Nanjing Medical University, Nanjing, China

**Keywords:** c-Met inhibitor, sorafenib, combination, hepatocellular carcinoma

## Abstract

Sorafenib, an oral multikinase inhibitor of Raf, VEGF and PDGF receptor signaling is approved for advanced hepatocellular carcinoma (HCC). One strategy to improve HCC therapy is to combine agents that target key signaling pathways. Aberrant mesenchymal-epithelial transition factor (c-Met) activation is associated with a variety of human malignancies and therefore represents a target for therapy. In this study, we investigated a novel c-Met inhibitor, DE605, together with sorafenib in hepatocellular carcinoma cells *in vitro* and *in vivo*. DE605 and sorafenib synergistically induced apoptosis in hepatocellular carcinoma cells. Mechanistically, DE605 activated the FGFR3/Erk pathway, which in turn was inhibited by sorafenib, resulting in synergism. Finally, DE605 and sorafenib significantly inhibited growth of PLC/PRF/5 hepatocellular carcinoma tumor xenografts in athymic nude mice. Importantly, no obvious weight loss (toxicity) was detected. Thus in combination, DE605 and sorafenib target complementary anti-apoptotic pathways and synergistically suppress HCC, providing the rationale for clinical studies with this novel combination.

## INTRODUCTION

Hepatocellular carcinoma (HCC) remains a major health problem worldwide as the third cause of cancer-related mortality and the primary cause of death among cirrhotic patients [1]. Hepatitis B and C, alcohol and aflatoxin have been identified as major risk factors leading to the development of HCC [2, 3]. Curative treatments, such as locoregional ablation, surgical resection, or liver transplantation, are only appropriate for a minority of patients with hepatocellular carcinoma, and their efficacies are limited by high recurrence rates. As most patients are diagnosed at an advanced disease stage, there is an urgent need for new systemic therapies [4]. Currently, sorafenib (Nexavar) is the only drug that has been approved by the U.S. Food and Drug Administration (FDA) for patients with advanced hepatocellular carcinoma. Sorafenib is an oral multikinase inhibitor that blocks various signaling pathways, including Raf kinases, VEGF, and platelet-derived growth factor receptors. In 2007, a pair of phase III studies indicated that sorafenib improved survival and the time to radiologic progression, leading to its approval for the treatment of advanced hepatocellular carcinoma [5, 6]. Sorafenib has also been approved for the treatment of advanced renal cell carcinoma, and recent preclinical studies have shown that it has broad-spectrum activity against models of several other human cancers, including melanoma, non–small cell lung cancer, colorectal cancer, and breast cancer [7]. Sorafenib executes its antitumor activities by targeting the Raf/Mek/Erk pathway, inducing cell apoptosis and blocking tumor angiogenesis [8]. In addition, recent evidence has indicated that Stat3 is a major kinase-independent target of sorafenib [9, 10].

The mesenchymal-epithelial transition factor (c-Met) is a receptor tyrosine kinase with hepatocyte growth factor (HGF) as its only known high-affinity ligand. During embryonic development, c-Met controls morphogenesis, invasiveness, and migration of precursor cells. In adult life, the protein is typically expressed at low levels in a range of tissues, predominantly involved in tissue repair, and activated by pathologic stimulation. More specifically, c-Met is essential in liver development and regeneration. In conditional c-Met knockout mice, liver repair is delayed or absent after hepatectomy or chemically induced liver injury [11]. In contrast, overexpression of HGF has been shown to increase liver regeneration and to cause significant liver enlargement after partial hepatectomy in mice [12]. However, c-Met expression is deregulated in many human malignancies, including hepatocellular carcinoma (HCC) [13]. In the cancer setting, c-Met/HGF mediates cellular proliferation, tumor invasion, and metastasis [14]. The underlying biologic mechanisms for the tumorigenicity of c-Met appear to involve the establishment of c-Met/HGF autocrine loops, overexpression of c-Met or HGF, and kinase-activating mutations in the c-Met gene [15]. Overexpression of c-Met alone has been demonstrated to be sufficient for developing HCC in Met-transgenic mice [16, 17]. In addition, c-MET overexpression is observed in 20-48% of human HCC samples [18-20] and where it may be a predictor for sensitivity to agents such as the tyrosine kinase inhibitor sorafenib [21].

Although systemic treatment with sorafenib is the recommended treatment in advanced HCC, its survival benefit is still limited, and novel tumor targets such as c-Met are warranted in this setting [22]. The use of c-Met inhibitors as a potentially viable treatment is supported by preclinical data showing that c-Met inhibition suppresses the growth of c-Met-positive HCC tumor cells [14, 23].

Given that hepatocellular carcinoma is a complex and heterogeneous tumor with aberrant activation of several signaling pathways, researchers have sought to target hepatocellular carcinoma with a combination of sorafenib plus chemotherapy or another targeted therapeutic agent [24-26]. This study was undertaken to evaluate the preclinical efficacy of the c-Met inhibitor, DE605, in combination with sorafenib in human hepatocellular carcinoma cells. We herein report, for the first time, that DE605 and sorafenib exhibited a synergistic interaction in killing hepatocellular carcinoma cells, inducing marked apoptosis via a caspase-dependent pathway. Our data suggest that the sorafenib-mediated inhibition of the DE605-activated fibroblast growth factor receptor 3 (FGFR3)/Erk signaling pathway may be a major component of the observed synergism. Moreover, we show that the combined treatment significantly decreases tumor volume in the PLC/PRF/5 xenograft model, compared with treatment by either drug alone. Taken together, these findings indicate that our combined treatment warrants further development for potential therapeutic applications in patients with hepatocellular carcinoma.

## RESULTS

### DE605 is a potent inhibitor of c-Met

DE605 was synthesized to specifically interact with c-Met and inhibit its kinase activity. The biochemical activity of this compound was measured in a flash-plate assay using recombinant human c-Met kinase domain and a biotinylated peptide substrate. Under these conditions, DE605 inhibited c-Met kinase activity with an average IC_50_ of 12.3 nM. To assess the selectivity of DE605 for c-Met kinase activity, this compound was profiled against a protein kinase panel of 242 human kinases. At 15.6 μM of DE605, a concentration approximately 1,200-fold above the IC_50_, DE605 showed an exceptionally high level of kinase selectivity toward c-Met with an inhibitory activity of more than 3,000-fold in comparison with the other 241 human kinases tested, as none of these kinases was inhibited by more than 50%.

**Figure 1:**
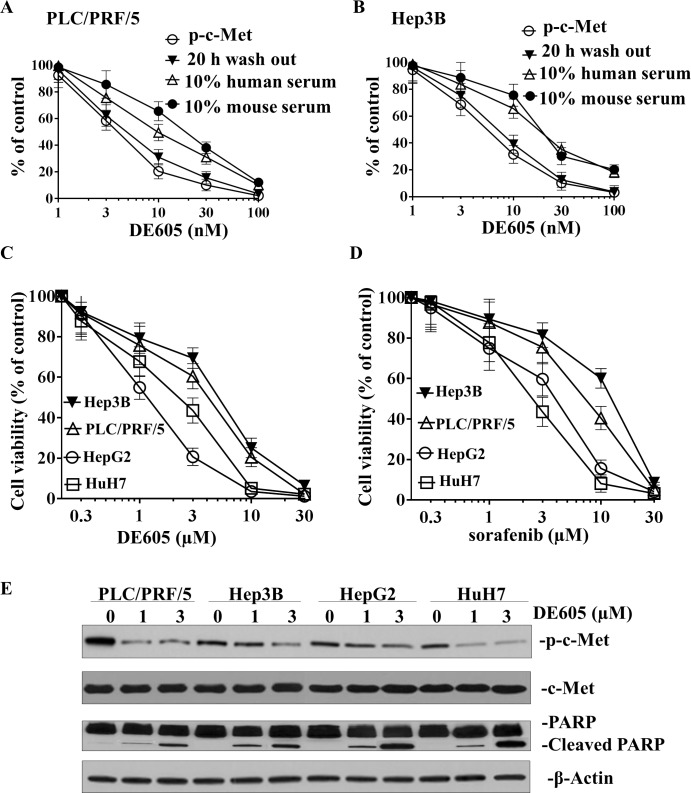
Effects of DE605 on c-Met phosphorylation, cell viability and epigenetic markers (A-B) PLC/PRF/5 (**A**) and Hep3B (**B**) hepatocellular carcinoma cells are characterized by c-Met expression and ligand dependence. *In vitro* PLC/PRF/5 and Hep3B culture was carried out under serum-free conditions or in the presence of 10% human or mouse serum. Upon HGF stimulation, inhibition of total c-Met phosphorylation by DE605 was assessed by c-Met capture ELISA using a pan-phospho-Tyr antibody. The levels of total phospho-c-Met were also assessed after wash-out of the inhibitors, as described in the Materials and Methods section. (**C-D**) Concentration-dependent effects of DE605 and sorafenib on cell viability in PLC/PRF/5, Hep3B, HepG2 and HuH7 hepatocellular carcinoma cell lines. The cells were treated with different concentrations of the indicated agents for 72 hours, and cell viability was measured by MTT assay. Each value represents the mean ± SD (n = 3). (**E**) Effects of DE605 on global changes of p-c-Met and PARP cleavage in four hepatocellular carcinoma cell lines. Cells were exposed to different concentrations of DE605 for 72 hours. Whole-cell lysates were collected and subjected to western blotting analysis.

We next investigated whether DE605 could inhibit c-Met phosphorylation induced in liver cancer cells by different mechanisms. To this end, we used the PLC/PRF/5 and Hep3B hepatocellular carcinoma cell lines, in which c-Met phosphorylation is respectively triggered by HGF binding or by c-Met gene amplification and ligand-independent activation. As shown in Fig. 1A and 1B, exposure of PLC/PRF/5 and Hep3B cells to DE605 resulted in inhibition of HGF-induced c-Met phosphorylation, with an average IC_50_ of 4.1 and 5.6 nM, respectively. Moreover, we defined the efficiency of the cellular uptake and retention of DE605. A series of wash-out studies was conducted, in which PLC/PRF/5 and Hep3B cells were incubated for 30 to 45 minutes in the presence of different concentrations of DE605, washed, stimulated with HGF, and subsequently assessed for c-Met phosphorylation. Our findings indicate that c-Met phosphorylation was inhibited upon exposure to DE605 and lasted for more than 20 hours in PLC/PRF/5 and Hep3B cells, with an average IC_50_ of 5.4 and 7.2 nM, respectively. These data show cellular retention of DE605, accompanied by sustained c-Met inhibition. Interestingly, the inhibitory effect of DE605 on HGF-induced c-Met phosphorylation was only moderately affected by the presence of 10% (v/v) murine or human serum, resulting in average IC_50_ values of 14.7 and 19.1 nM, respectively. Higher serum concentrations could not be used in this test, as they suppressed the HGF-induced c-Met phosphorylation, probably because of HGF binding to serum proteins or HGF inactivation by serum proteases. Taken together, our data indicate that DE605 is a potential and reversible inhibitor of c-Met.

### Effects of DE605 and sorafenib on cell viability in hepatocellular carcinoma cells

The effects of DE605 and sorafenib on cell viability in four hepatocellular carcinoma cell lines were determined using a MTT assay. PLC/PRF/5, Hep3B, HepG2 and HuH7 Cells were treated with different concentrations of DE605 (0, 0.3, 1, 3, 10 and 30 μM) and sorafenib (0, 0.3, 1, 3, 10 and 30 μM) for 72 h. As shown in Fig. 1C, DE605 was able to repress cell growth in all four cell lines in a dose-dependent manner, with IC_50_ values of 3.4, 4.5, 1.2 and 2.0 μM, respectively. Interestingly, the cell lines exhibited differential sensitivities to the cytotoxic effects of sorafenib. HepG2 and HuH7 were sensitive to sorafenib, with IC_50_ values of 3.3 and 2.4 μM, respectively. Whereas, PLC/PRF/5 and Hep3B were more resistant, with IC_50_ values of 6.7 and 10.5 μM, respectively (Fig. 1D). In order to determine the side effects of DE605 on normal cells, HL-7702 normal liver cells were treated with DE605 or sorafenib. We obtained that DE605 and sorafenib exhibited similar cytotoxicity in HL-7702 normal liver cells. However, DE605 exhibited a little lower cytotoxicity in HL-7702 cells than in HCC cells (supplemental Fig.S2A). Here, we further confirmed the epigenetic effects of DE605 by western blotting analysis of c-Met, and apoptotic markers in hepatocellular carcinoma cells. As shown in Fig. 1E, DE605 inhibited the phosphorylation of c-Met expression in all four cell lines. This was accompanied by the induction of cleavage of PARP. Collectively, our results indicate that both DE605 and sorafenib exhibited cytotoxic effects in hepatocellular carcinoma cell lines in a dose-dependent manner.

### Synergistic interaction between DE605 and sorafenib in hepatocellular carcinoma cells

**Figure 2:**
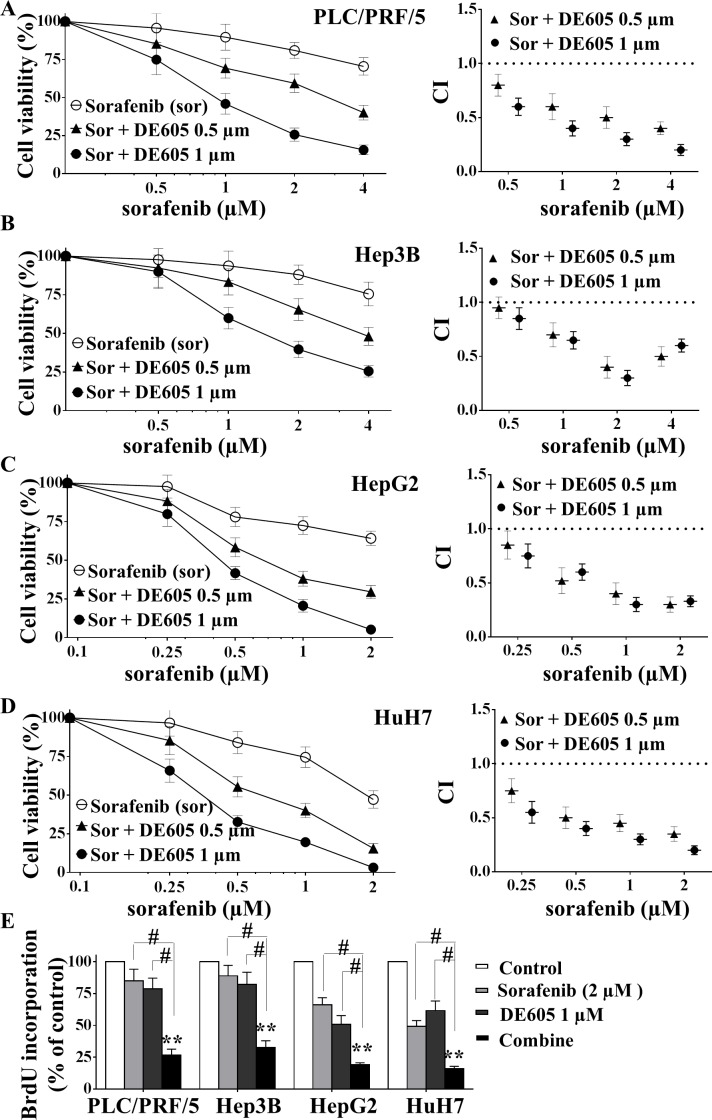
Effects of sorafenib in combination with DE605 on cell viability and proliferation in hepatocellular carcinoma cells PLC/PRF/5 (**A**), Hep3B (**B**), HepG2 (**C**) and HuH7 (**D**) cells were treated with various concentrations of sorafenib in combination with DE605 for 72 hours, and cell viability was measured by MTT assay (left). The combination index (CI) values were calculated by CompuSyn software (right). CI values <1 represent synergism, and the numbers reflect the corresponding data points (left). (**E**) Cells were treated with indicated agents for 72 hours, BrdU was added during last 2 h of incubation period and the assay was performed by using a Proliferation Assay kit according to the manufacturer's instructions (Millipore, Billerica, MA). Each value represents the mean ± SD (n = 3). **, *P* < 0.01 compared with the control group. #, *P* < 0.01, compared with the sorafenib or DE605 group.

To investigate the effect of the combined treatment in experimental models of HCC, four hepatocellular carcinoma cell lines were treated with different concentrations of sorafenib in the presence or absence of DE605 for 72 hours, and cell viability was determined by MTT assay. Our results revealed that DE605 significantly and concentration-dependently enhanced sorafenib-mediated cytotoxicity in PLC/PRF/5 cells (Fig. 2A). To explore whether the combined treatment had a synergistic impact on cell viability, the combination index values of each dose were calculated by the CompuSyn software. The results revealed that DE605 exhibited a synergistic effect in combination with sorafenib at low concentrations (0.5 and 1.0 μM) (Fig. 2A, right). In addition, to confirm these results, PLC/PRF/5 cells were treated with sorafenib and a previously reported c-MET selective inhibitor tivantinib (ARQ 197) [27] for 72 hours, cell viability was determined by MTT assay and combination index values was calculated. Interestingly, the results revealed that tivantinib also exhibited a synergistic effect in combination with sorafenib at low concentrations (0.25 and 0.5 μM), which is similar to those obtained in sorafenib combination with DE605 (supplemental Fig. S2B). Moreover, the synergistic effect between DE605 and sorafenib were also observed in Hep3B, HepG2 and HuH7 cells, indicating that this was not a cell line–specific effect (Fig. 2B, 2C and 2D). Furthermore, the combined treatment significantly enhanced the antiproliferative effects in all four tested cell lines as evidenced by BrdU incorporation assay (Fig. 2E). Taken together, our results indicate that DE605 combination with sorafenib synergistically increased cytotoxicity and improved the anti-proliferative effects consistently in different hepatocellular carcinoma cell lines.

### DE605 in combination with sorafenib enhances apoptotic cell death via a caspase-dependent pathway

**Figure 3:**
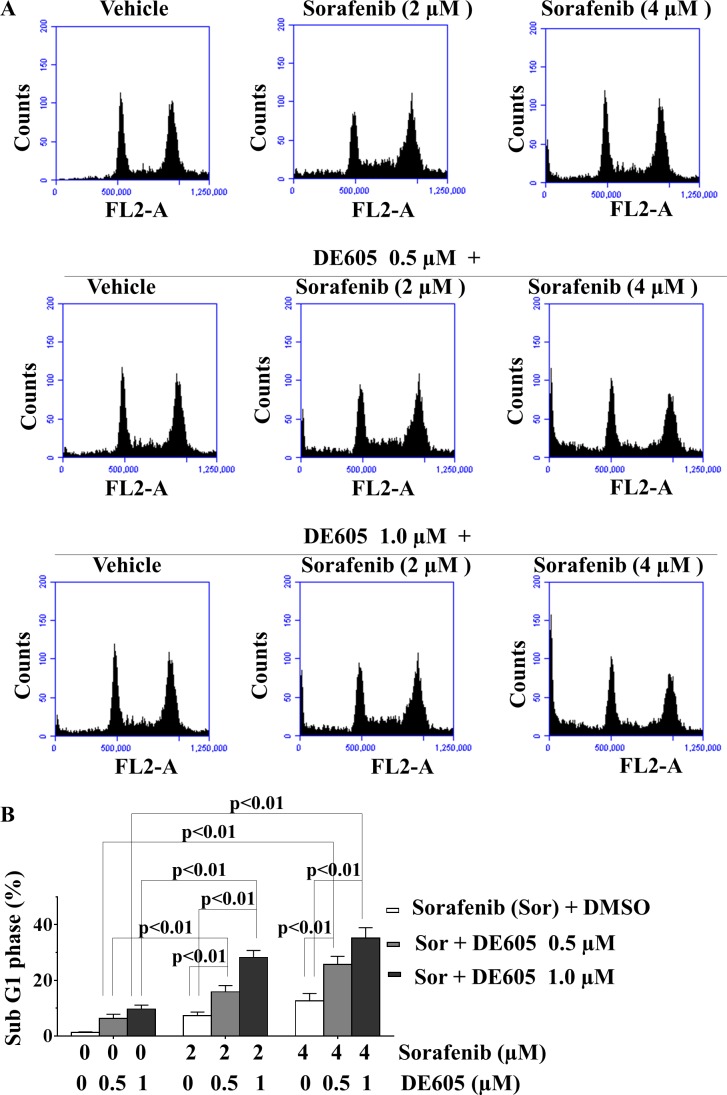
Effects of sorafenib in combination with DE605 on cell-cycle progression in PLC/PRF/5 cells (**A**) PLC/PRF/5 cells were treated with the indicated drugs for 72 hours, and cell-cycle distribution was analyzed by flow cytometry. (**B**) Statistical analysis of sub-G_1_ phase in PLC/PRF/5 cells exposed to DMSO, sorafenib alone, DE605 alone, or in sorafenib/DE605 combination. Data, mean ± SD (*n* = 3).

As sorafenib and c-Met inhibitors reportedly induce apoptosis in cancer cells [8, 28], we examined the impact of our drug combination on programmed cell death. First, the effect of co-administration of DE605 and sorafenib on cell-cycle distribution was analyzed by flow cytometric analysis. As shown in Fig. 3A, no appreciable cell-cycle arrest was observed after sorfenib or DE605 treatment alone. However, the combined treatment significantly enhanced the percentage of sub-G_1_ phase cells over that seen in cells treated with either drug alone, suggesting that apoptosis is the main cause of cell death in the co-treated PLC/PRF/5 cells (Fig. 3B).

**Figure 4:**
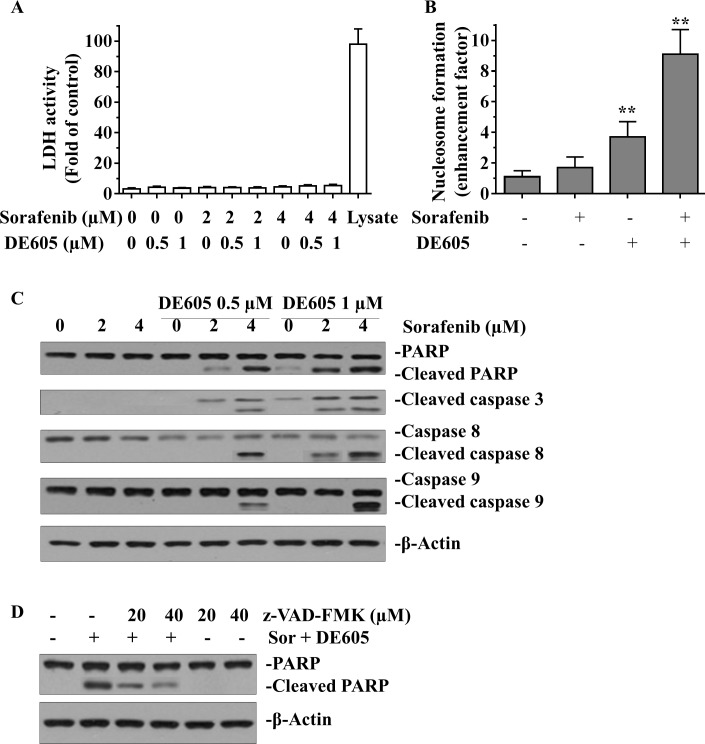
Effect of sorafenib plus DE605 on LDH activity, nucleosome formation and the apoptosis-related proteins in PLC/PRF/5 cells (**A**) PLC/PRF/5 cells were exposed to indicated drugs for 72 hours. LDH acticity in cell supernatants or lysates was analyzed by the CytoTox 96® Non-Radioactive Cytotoxicity Assay (Promega, Madison, WI) according to manufacturer's protocol. (**B**) PLC/PRF/5 cells were treated with sorafenib (4 μM) alone or in combination with DE605 (1 μM) for 72 hours. Nucleosome formation was measured using the Cell Death ELISA Kit (Roche Applied Science). Data, mean ± SD (*n* = 3; **, *P* < 0.01 compared with the control group). (**C**) Cells were treated with various concentrations of sorafenib in combination with DE605 for 72 hours. (**D**) Cells were exposed to sorafenib (Sor, 4 μM) in combination with DE605 (1 μM) in the presence or absence of zVAD–FMK for 72 hours. Whole-cell lysates were collected and subjected to Western blot analysis with the indicated antibodies.

Meanwhile, the possibility of necrotic effect was excluded by examining LDH leakage in the supernatants of drug-treated PLC/PRF/5 cells (Fig. 4A). To confirm this effect, nucleosome formation (representing DNA fragmentation) was determined in drug-treated cells. The results showed that DE605 dramatically enhanced sorafenib-induced nucleosome formation in PLC/PRF/5 cells (Fig. 4B).

We further investigated the underlying mechanism of co-treatment-induced apoptosis in PLC/PRF/5 cells by western blotting analysis. As shown in Fig. 4C, when cells were co-treated with sorafenib and DE605 at concentrations lower than the IC_50_ value, we observed cleavage of PARP and caspase-3, indicating the occurrence of apoptosis. Moreover, caspase-8 and caspase-9 were also activated by the combined treatment, indicating that both intrinsic and extrinsic apoptotic pathways are involved in this phenomenon. Furthermore, co-treatment with the pan-caspase inhibitor, z-VAD–FMK, attenuated PARP cleavage in a concentration-dependent manner, suggesting that the observed apoptosis was triggered via a caspase-dependent pathway (Fig. 4D). Overall, these data indicate that the synergism between sorafenib and DE605 in killing hepatocellular carcinoma cells might be achieved through caspase-dependent induction of apoptosis.

### DE605 plus sorafenib alters multiple signaling pathways in hepatocellular carcinoma cells

**Figure 5:**
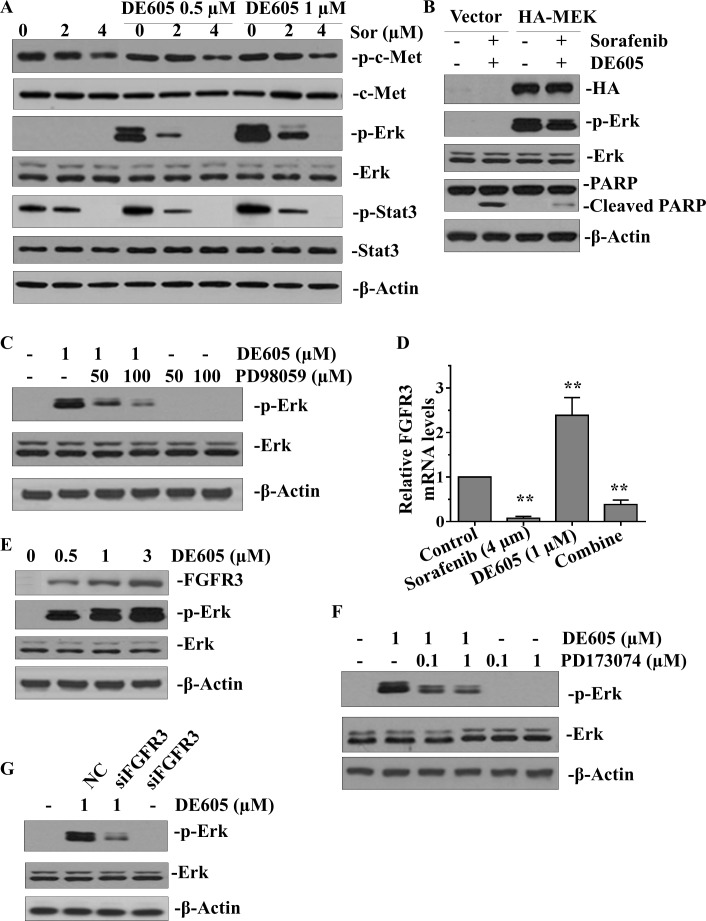
Effects of sorafenib plus DE605 on multiple signaling pathways in PLC/PRF/5 cells (**A**) PLC/PRF/5 cells were treated with various concentrations of sorafenib alone or in combination with DE605. (**B**) PLC/PRF/5 cells were transfected with control vector or HA–Mek for 24 hours and then exposed to sorafenib (4 μM) in combination with DE605 (1 μM) for 72 hours. (**C**) Cells were exposed to DE605 in the presence or absence of PD98059 for 72 hours. Whole-cell lysates were collected and subjected to western blotting analysis (A–C). (D–G) Cells were treated with indicated agents for 72 hours or transfected with FGFR3 siRNA. Relative mRNA levels of FGFR3 were determined by RT-PCR (**D**). Data, mean ± SD (*n* = 3; **, *P* < 0.01 compared with the control group). Whole-cell lysates were subjected to western blotting analysis (**E**, **F** and **G**).

To gain insights into the mechanisms underlying the synergistic interaction between sorafenib and DE605 in PLC/PRF/5 cells, we examined whether the combined treatment enhanced the signaling pathways affected by each agent alone. As shown in Fig. 5A, there was no profound enhancement of c-Met following the combined treatment. Therefore, the observed synergistic effects may not result from augmentation of the epigenetic effects triggered by DE605. Sorafenib has been shown to inhibit the Raf/Mek/Erk pathway, and patients with hepatocellular carcinoma with higher levels of phosphorylated Erk have a better survival rate [8, 29]. Furthermore, Stat3 was recently reported to be a major kinase-independent target of sorafenib [10]. Therefore, we postulated that the combination of DE605 and sorafenib might impact these signaling pathways. Interestingly, our data show that DE605 alone dramatically elevated the level of phosphorylated-Erk (p-Erk) and increased its downstream signaling, as reflected by increases in phosphorylated-Stat3-Ser^727^ (p-Stat3). These effects were concentration-dependently abrogated by sorafenib (Fig. 5A). Moreover, forced expression of constitutively active Mek attenuated the co-treatment-induced PARP cleavage, suggesting that the sorafenib-mediated inactivation of Erk may play a pivotal role in DE605-mediated apoptosis (Fig. 5B). In addition, pharmacologic inhibition of Mek by PD98059 attenuated DE605-induced p-Erk (Fig. 5C), confirming the importance of Erk activation in the synergistic interaction between DE605 and sorafenib. We further investigated the mechanism behind Erk activation by cDNA microarray to examine differential expressed genes affected by DE605 (1.0 μM) in PLC/PRF/5 cells. The result showed that the mRNA level of FGFR3 was up-regulated by DE605, but this phenomenon was abrogated in the presence of sorafenib (Fig. 5D). Moreover, the protein levels of FGFR3 and p-Erk were also up-regulated by DE605 in a concentration-dependent fashion (Fig. 5E). Furthermore, co-administration of FGFR3 inhibitor (PD173074) or silencing of FGFR3 attenuated DE605-induced Erk phosphorylation, suggesting that transcriptional activation of FGFR3 may contribute to Erk activation in PLC/PRF/5 cells (Fig. 5F and 5G). Furthermore, in order to provide evidences that the mechanism behind Erk activation is not specific to PLC/PRF/5 cells, we employed another hepatocellular carcinoma cell line Hep3B for further experiments. The results showed that the mRNA level of FGFR3 was up-regulated by DE605 and abrogated in the presence of sorafenib in Hep3B cells (supplemental Fig. S3A). The protein levels of FGFR3 and p-Erk were also up-regulated by DE605 in a concentration-dependent fashion (supplemental Fig.S3B). Co-administration of FGFR3 inhibitor (PD173074) or siFGFR3 attenuated DE605-induced Erk phosphorylation in Hep3B cells (supplemental Fig. S3C and S3D). These results further confirm the evidences in PLC/PRF/5 cells that the mechanism is not specific to PLC/PRF/5 cells. Collectively, these data suggest that the synergistic interaction between DE605 and sorafenib is achieved at least partially via inhibition of the FGFR3/Erk signaling pathway.

### DE605 plus sorafenib inhibits tumor xenograft growth in athymic nude mice

**Figure 6:**
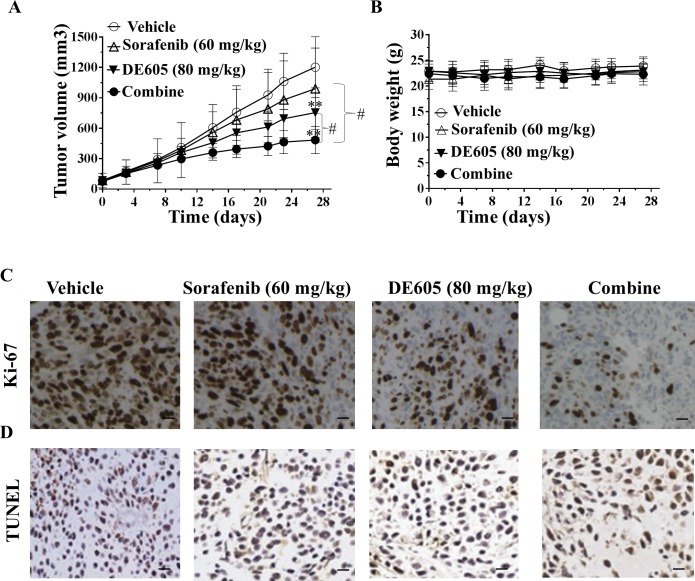
Antitumor activity of DE605 plus sorafenib in a PLC/PRF/5 xenograft model Athymic nude mice bearing subcutaneously established PLC/PRF/5 xenograft tumors were randomized to four groups (*n* = 7), and received the indicated treatments by gavage. (**A**) tumor volumes were measured twice per week and the curves of tumor growth volume was expressed as mean ± SD. **, *P*< 0.01 compared with the control group, #, *P*< 0.01, compared with the sorafenib or DE605 group. (**B**) body weights were measured twice per week, and are expressed as mean ± SD. (**C-D**) IHC analysis of intratumoral proliferation in PLC/PRF/5 xenograft tumors. Tumors were harvested and paraffin-embedded tumor tissues were subjected to immunostaining for Ki-67 (**C**) and TUNEL (**D**). Images were captured by Zeiss Axioskop-2 microscope under 200× magnification. Scale bar, 100 μm.

To evaluate whether the synergistic effect of DE605 plus sorafenib could be clinically relevant, we examined the antitumor activity of this cotreatment in athymic nude mice bearing established PLC/PRF/5 tumor xenografts. As seen in Fig. 6A, Oral treatment with sorafenib or DE605 alone for 28 days resulted in a modest tumor growth inhibition (TGI) in the nude mice (17.3% and 37.3%, respectively) compared with the control group. DE605 in combination with sorafenib significantly suppressed tumor growth, with TGI of 59.7%. In addition, the cotreatment exhibited significant difference compared with sorafenib or DE605 alone in the PLC/PRF/5 xenograft model. Moreover, the average body weights of the mice were comparable throughout the experimental period. As seen in Fig. 6B, there was no difference in body weight in treatment groups compared with the control group. These data suggest that the treatment in these studies is not associated with apparent gross toxicity.

### DE605 plus sorafenib inhibits proliferation with induction of apoptosis in tumor tissues

To correlate the *in vivo* antitumor effects with the mechanisms identified *in vitro*, intratumoral biomarkers were assessed by immunohistochemical analysis. Uncontrolled tumor cell proliferation is a characteristic feature of most cancers. We therefore analyzed the hepatocellular carcinoma tumor xenografts for the potential anti-proliferative effects of DE605 plus sorafenib using immunohistochemical detection of Ki-67-positive cells. As shown in Fig. 6C, co-administration of DE605 and sorafenib significantly decreased the expression of Ki-67, a cell proliferation marker. In addition, to determine whether inhibition of tumor growth by administration DE605 plus sorafenib is caused by the apoptosis of tumor cells in xenograft tissues, the apoptotic effect of DE605 plus sorafenib on hepatocellular carcinoma tumor tissues was identified by expression of the DNA fragment by TUNEL assay. The results showed that greater numbers of TUNEL-positive cells in the samples from co-treatment as compared with the numbers in the samples from the non-treated tumors (Fig. 6D). Taken together, these data are consistent with our *in vitro* data, indicating that co-administration with DE605 and sorafenib significantly enhanced the antitumor activity *in vivo*.

## DISCUSSION

Human hepatocellular carcinoma (HCC) remains a major health problem, as it is the sixth most common cancer and the third most common cause of cancer-related deaths worldwide [1]. There are multiple therapeutic options for this disease, resection and transplantation are the only curative treatments available but are greatly hampered by high recurrence rates [3]. HCC is a complex and heterogeneous tumor that has been associated with genomic aberrations. The key signal transduction pathways that have been implicated in the pathogenesis of hepatocellular carcinoma are the EGFR, Ras/Raf/Mek/Erk, phosphoinositide 3-kinase/Akt, mTOR, HGF/c-Met, Wnt, and Hedgehog signaling cascades [24]. Drugs that selectively target these molecules might, therefore, have therapeutic potential.

Currently, the multi-kinase inhibitor sorafenib is the only FDA-approved treatment for patients with advanced disease, necessitating the development of novel compounds that are effective against this devastating disease [6, 30]. However, sorafenib mainly delays the time to radiologic progression rather than inducing tumor regression. Therefore, new approaches with improved therapeutic efficacy are urgently needed. A number of treatment strategies have been developed for patients who do not respond to or tolerate sorafenib, including the use of monoclonal antibodies (e.g., ramucirumab) and tyrosine kinase inhibitors (e.g., erlotinib, sunitinib, vandetanib, cediranib, brivanib, foretinib, and dovitinib) [4]. In addition, combined treatment with sorafenib plus another agent, such as erlotinib, everolimus, or CS-1008, is currently being investigated [31]. Nonetheless, few of them are testing the efficacy of a c-Met inhibitor combination with sorafenib in this difficult-to-treat cancer. In the present study, we provide compelling evidence that DE605, a novel potent inhibitor of c-Met, combined treatment with sorafenib significantly inhibits the growth of human hepatocellular carcinoma cells *in vitro* and *in vivo*.

The rationale behind developing c-Met kinase inhibitors for the treatment of cancer is based on multiple lines of preclinical evidence showing that aberrant activation of the c-Met/HGF signaling pathway plays a pivotal role in cancer progression and metastasis by promoting cell proliferation, survival, and motility [32]. Dysregulation of the c-Met/HGF pathway can occur by ligand-dependent or -independent mechanisms. Indeed, overexpression of wild-type c-Met or engagement by HGF in an autocrine or paracrine fashion induces tumor transformation [33, 34]. Thus, c-Met are promising drug targets for the treatment of hepatocellular carcinoma.

Small-molecule kinase inhibitors have a broad therapeutic efficacy and their development is becoming increasingly feasible as a result of the improved understanding of the structure and molecular mode of action of kinases. Based on these studies and increasing preclinical evidence, a number of c-Met inhibitors are currently under study in several clinical trial phases [27]. In addition to being tested as a single therapeutic agent, c-Met inhibitors are also being assessed in combination with chemotherapy agents [35, 36]. Our preclinical data show that DE605 block constitutive phosphorylation of c-Met, thereby interfering with survival of susceptible tumor cells. Such observations are in agreement with reports on the effects of JNJ-38877605, INCB28060, EMD 1214063 and EMD 1204831, which also belong to the category of type I c-Met inhibitors [37, 38].

Our *in vitro* study shows that treatment with sorafenib in PLC/PRF/5 and Hep3B cells have higher IC_50_ than HepG2 and HuH7 cells. These data reinforce the results reported earlier, which showed PLC/PRF/5 and Hep3B are most resistant to sorafenib among different liver cancer cell lines [39, 40]. On the basis of these findings, we used PLC/PRF/5 cells in most of the mechanistic studies to elucidate the synergistic effects of DE605 and sorafenib in hepatocellular carcinoma. In the present study, we observed a synergistic interaction between DE605 and sorafenib in human hepatocellular carcinoma cells. Therefore, DE605 not only has a broad spectrum of action against different types of cancer, it also offers a new tool for treating hepatocellular carcinoma when combined with sorafenib.

From a therapeutic point of view, it remains controversial as to whether single- or multiple-targeted inhibitors are most advantageous. Clinical evidence suggests that multiple-targeted inhibitors may be more often associated with dose-limiting effects, whereas single-targeted kinase inhibitors can be used at maximal dosing level without causing toxic effects. Indeed, XL880 and XL184, which target multiple, non-family-related kinases including VEGF receptor, are associated with dose-limiting toxicities that may not be attributed to c-Met inhibition [37]. On the other hand, the achievement of therapeutic effects with DE605 and sorafenib may require thorough patient screening to identify a responsive subset, in which the tumor is sensitive to selective disruption of the c-Met signaling pathway.

Sorafenib executes its antitumor activities, which include triggering cell apoptosis and blocking tumor angiogenesis, by targeting the Raf/Mek/Erk pathway. A previous study showed that patients with high levels of p-Erk have a greater survival rate [8]. It was reported that treatment with c-met inhibitor leads to inhibit phosphorylation of ERK, down-stream signals of c-Met [41, 42]. However, the functional role of the activation of Erk kinase is often controversially discussed. By contrast, Liu et al. [43, 44] showed that inhibition of c-Met was associated with ERK activation in human lung cancer A549 cells. Of special interest was the observation that DE605, at the concentrations below IC_50_ value, dramatically activated Erk and aspects of its downstream signaling, such as p-Stat3-Ser^727^. DE605-mediated Erk activation was concentration-dependently abrogated by sorafenib. Ectopic expression of constitutively active Mek reversed the apoptotic cell death triggered by the co-treatment in PLC/PRF/5 cells. Furthermore, pharmacologic inhibition of Mek attenuated DE605-induced p-Erk. Therefore, we postulated that treatment with low concentrations of DE605 may render hepatocellular carcinoma cells more dependent on Erk signaling and increase their sensitivity to sorafenib. Our observations are in accordance with previous studies showing that interruption of Erk signaling by Mek inhibitors sensitized tumor cells to kinase inhibitor–induced apoptosis [39, 45-47].

In the present study, we observed the transcriptional activation of FGFR3 by DE605, and that was abrogated in the presence of sorafenib. DE605-induced Erk activation was abrogated by FGFR3 inhibitor (PD173074), suggesting that FGFR3 induction may be the underlying mechanism of Erk phosphorylation. Such observations are in agreement with reports on the effects of panobinostat and MPT0E028 [39, 48]. In this study, we provide compelling evidence that combined treatment with the c-Met inhibitor, DE605, plus sorafenib synergistically inhibits the growth of human hepatocellular carcinoma cells *in vitro*. Moreover, we further reinforce the data by elucidating the underlying mechanisms by which sorafenib enhances DE605-mediated apoptosis by inhibiting the FGFR3/Erk signaling pathway.

Although *in vitro* cell culture models are a good system for preliminary screening of the effects of anti-tumor agents; the observations must be verified *in vivo* using animal models prior to their potential consideration of their use in humans. We therefore used an *in vivo* model of xenografts of PLC/PRF/5 tumor cells in athymic nude mice to verify the anti-tumor potential of DE605 combination with sorafenib against liver tumor cell growth. Our study provides evidence that administration of DE605 plus sorafenib significantly inhibits the growth of PLC/PRF/5 liver tumor xenografts without any apparent sign of toxicity in the athymic nude mice. These data are in accordance with the decreased proliferation documented by Ki67 immunostaining. Additionally, DE605 plus sorafenib induced apoptosis as indicated by TUNEL staining in tumor tissues. These *in vivo* results are similar to our *in vitro* study, which further suggests that DE605 combination with sorafenib may be an applicable approach to enhance the antitumor activity against liver cancer. In contrast, Bladt et al. [49] reported that the c-Met inhibitor MSC2156119J (EMD 1214063) combination with sorafenib did not improve efficacy in MHCC97H human HCC xenograft model and HuPrime model with human HCC explants. Therefore, different results may be showed in different c-Met inhibitor and xenograft tumor models.

In conclusion, the present study demonstrates that DE605 and sorafenib interact in a synergistic manner to invoke strong anticancer activity against hepatocellular carcinoma by inducing apoptosis as well as inhibiting cell growth/proliferation. Moreover, we also show that sorafenib-mediated inhibition of DE605-induced Erk activation might play a pivotal role in this synergistic effect. These results suggest that combining DE605 with the standard of care sorafenib could be an attractive strategy for treating patients with advanced hepatocellular carcinoma.

## MATERIALS AND METHODS

### Antibodies and reagents

Antibodies were obtained from the following commercial sources: PARP, caspase-3, caspase-8, caspase-9, phospho-Erk, and Erk (Cell Signaling Technology); p-c-Met, c-Met, and FGFR3 (Santa Cruz Biotechnology); pan-actin (Millipore); phospho-stat3-Ser^727^ and Stat3 (BD Biosciences). propidium iodide, MTT, z-VAD–FMK, PD98059, PD173074, and all of the other chemical reagents were obtained from Sigma. RPMI-1640 medium, Dulbecco's Modified Eagle Medium (DMEM), FBS, penicillin, streptomycin, and all other tissue culture regents were obtained from GIBCO/BRL Life Technologies. Sorafenib (purity ≥ 99%) was purchased from Biovision. Tivantinib (ARQ 197) was purchased from ChemieTek. For *in vitro* administration, sorafenib, tivantinib or DE605 (structure and scheme of DE605 synthesis shown in supplemental Fig. S1) were dissolved in DMSO (Sigma) to a concentration of 10 mM and further diluted to appropriate final concentration in RPMI 1640 with 10% fetal bovine serum. DMSO in the final solution did not exceed 0.1% (v/v). For *in vivo* testing, sorafenib or DE605 were dissolved in Cremophor EL/ethanol (50:50; Sigma Cremophor EL, 95% ethanol) at 4 × concentration. This 4 × solution was prepared fresh every 4 days. Final dosing concentration was prepared by diluting the 4 × solution to 1× with sterile water. The 1× solution was prepared just before it was given to the mice.

### Cell culture

Human hepatocellular carcinomacell lines, PLC/PRF/5, Hep3B, HepG2 and HuH7, as well as normal liver cell line HL-7702 were purchased from American Type Culture Collection (Rockville, MD), and cultured as recommended as monolayers in RPMI-1640 supplemented with 10% heat-inactivated fetal bovine serum (Hyclone, Logan, UT), penicillin (100 U/ml)/streptomycin (100 μg/ml)/amphotericin B (0.25 μg/ml) from Invitrogen (Carlsbad, CA) in a humidified incubator at 37°C in a 5% CO_2_ atmosphere.

### c-Met *in vitro* kinase assay

Kinase inhibition by DE605 was assessed *in vitro* using a panel of 242 different kinases. Biochemical activity was measured in a flash-plate assay. His6-tagged recombinant human c-Met kinase domain (Aa 974–end; 20 ng) and biotinylated poly-Ala-Glu-Lys-Tyr (6:2:5:1; 500 ng) were incubated with or without the test compound for 90 minutes at room temperature in 100 μl buffer containing 0.3 μCi ^33^P-ATP, 2.5 μg polyethylene glycol 20.000, and 1% dimethyl sulfoxide (DMSO), as previously described [50]. Radioactivity was measured with a TopCount microplate scintillation and luminescence counter (Packard BioScience BV). Inhibitory 50% concentration values (IC_50_) were calculated by nonlinear regression analysis using the RS/1 software program.

### Phospho-c-Met-capture ELISA

Total c-Met phosphorylation was assessed by c-Met–capture ELISA in Nunc-Immuno MicroWell 96-well solid plates (Sigma-Aldrich) as previously described [37]. PLC/PRF/5 human hepatocellular carcinoma cells were seeded 2 days before treatment, serum-starved for 20 hours, and treated on day 3 with different concentrations of DE605 or 0.1% DMSO for 45 minutes at 37°C, 5% CO_2_. Upon stimulation with 100 ng/ml HGF for 5 minutes, cells were lysed with 70 μl per well ice-cold lysis buffer [20 nmol/l HEPES, pH 7,4; 10% (V/V) Glycerol; 150 nmol/l NaCl; 1% (V/V) Triton-X-100; 2 nmol/l EDTA] supplemented with protease and phosphatase inhibitors. In the wash-out experiments, PLC/PRF/5 were treated with DE605 for 45 minutes, washed, and incubated in serum-free medium for 14 hours, before stimulation with HGF (100 ng/ml). In the ELISA, the capture antibody was specific for the c-Met extracellular domain, whereas an anti-phosphotyrosine biotin-labeled antibody was used for detection. Tyrosine phosphorylation was revealed using a streptavidin peroxidase conjugate and chemiluminescence read-out.

### MTT assay

Cells were seeded in 96-well plates (3,000 cells/well) and incubated overnight for attachment, and were then treated with indicated agents in 10% FBS-supplemented medium for 72 hours. The medium was replaced with MTT (0.5 mg/ml) at 37°C for 2 hours. After removal of medium, the cells were lysed with 200 μl per well dimethyl sulfoxide (DMSO), and absorbance at 570 nm was measured and the values of 50% inhibition concentration (IC_50_) for each drug were determined. Cell viability was evaluated by measuring the optical density at 570 nm using an Automated Microplate Reader (Bio-Tek, USA). The percentage of viable cells was calculated using the following formula: cell viability (%) = (OD of treated cells/OD of control cells) × 100. All assays were performed in triplicate with at least 3 independent experiments. The combination index value was determined from the fraction-affected value of each combination according to the Chou–Talalay method by using CompuSyn software (ComboSyn, Inc.), and a combination index value below 1 represents synergism [51].

### Cell proliferation assay

Cell proliferation was assessed by bromodeoxyuridine (BrdU) incorporation assay using the Proliferation Assay Kit (Millipore). Cells were seeded in 96-well plates (5,000 cells/well) with 10% FBS in culture medium, and treated with indicated agents for 72 hours. BrdU was added during last 2 hours of incubation period. The assay was performed according to the manufacturer's instructions.

### Flow cytometric analysis

After drug treatment, the cells were harvested by trypsinization, washed with PBS, then pellets were resuspended and fixed in ethanol (70%, v/v) at −20°C overnight, and washed once with PBS. After centrifugation, the cells were incubated for 15 minutes at room temperature in 0.1 ml of phosphate-citric acid buffer. Cells were stained with propidium iodide staining buffer containing Triton X-100 (0.1%, v/v), RNase A (100 μg/ml), and propidium iodide (80 μg/ml) for 30 minutes in the dark. Cell-cycle distribution was analyzed by flow cytometry with CellQuest software (Becton Dickinson).

### Transient transfection and Western blot analysis

The cells were transfected with lipofectamine 2000 (Invitrogen) according to the manufacturer's protocol. Silencer select siRNA against ERK was purchased from Ambion. Plasmid expressing constitutively active Mek (MEK-specific primer is 5′-ACCTTGAATACCACTCC-3′) was prepared as described previously [52]. FGFR3 siRNAs and nonsense siRNA used as a control were chemically synthesized (Ambion, Huntingdon, UK). The sequences of the sense strands were as follows: control siRNA, 5′-GGCAAGAUUCUUCUCGUUGTT-3′; FGFR3 siRNA, 5′-GCCUUUACCUUUUAUGCAATT-3. The expression and phosphorylation of c-Met and downstream signaling factors were evaluated by Western blotting. Briefly, cells were harvested, and lysed with ice-cold lysis buffer supplemented with protease inhibitors. Equivalent amounts of protein (30 μg) from each cell lysate were resolved on SDS-PAGE. Gels were electroblotted onto nitrocellulose membranes (0.45 μM; Bio-Rad), which were then incubated in blocking solution (1×PBS, 0.1% Tween-20, 5% non-fat dry milk powder) for 1 h at room temperature. Membranes were incubated with the primary antibody at 4°C overnight. After additional TBST washes, membranes were incubated with corresponding horseradish peroxidase-conjugated secondary antibodies (Bio-Rad) for 1 h at room temperature and detected by the enhanced chemiluminescence method (SuperSignal West Pico substrate; Pierce; Rockford, IL).

### Real time qRT-PCR analysis

Cells were harvested, and total RNA was extracted in Trizol (Invitrogen; Carlsbad, CA) according to the manufacturer's protocol. The first-strand cDNA was synthesized using 1.0 μg total RNA and the iScript^TM^Reverse Transcription Supermix for real-time polymerase chain reaction (RT-PCR) (Bio-Rad). PCR was performed in triplicate using the SsoFast^TM^ Probes Supermix (Bio-Rad)in a final reaction volume of 10 μl with gene-specific primer/probe sets, and a standard thermal cycling procedure(40 cycles) on a Bio-Rad CFX96^TM^ Real-Time PCR System. RNA levels of FGFR3 and 18S were assessed using the TaqMan Gene Expression real-time PCR assays. Primer sequences of FGFR3 are 5′-ACAGCTCAGCTCCACAGCAT-3′ (forward) and 5′-GAGTCCTTGGGGACGGAG-3′ (reverse). Results were expressed as the threshold cycle (Ct). Relative quantification of target transcripts was determined by the comparative Ct method (ΔΔCt) according to the manufacturer's protocol. Relative gene expression was normalized to 18S and calculated by using the 2^−ΔΔCt^ method [53]. Control PCR experiments in the absence of reverse transcription were performed to confirm that the total RNA was not contaminated with genomic DNA.

### LDH assay

Cells were treated with drugs at indicated concentrations for 72 hours followed by the CytoTox 96 Non-Radioactive Cytotoxicity Assay (Promega) at 490 nm to measure the levels of LDH (lactate dehydrogenase) release according to the manufacturer's protocol.

### Tumor xenograft model

This study was carried out in strict accordance with the recommendations in the Guide for the Care and Use of Laboratory Animals of the National Institutes of Health. The protocol was approved by the Committee on the Ethics of Animal Experiments of Wuxi People's Hospital. The IACUC committee members at Wuxi People's Hospital approved this study. All surgery was performed under sodium pentobarbital anesthesia, and all efforts were made to minimize suffering. Female athymic nude mice of 4–5 weeks of age were purchased from Shanghai SLAC Laboratory Animal Co., Ltd. (Shanghai, China) and were housed in the Animal Resource Facility. To determine the antitumor activities of DE605 plus sorafenib *in vivo*, exponentially growing PLC/PRF/5 cells (1.0×10^7^ in 100 μl PBS) were injected subcutaneously in the right flank of each mouse. As tumors became established, mice were randomized to four groups that received the following agents by gavage: (A) vehicle, (B) sorafenib (60 mg/kg, po, qd), (C) DE605 (80 mg/kg, po, qd), and (D) DE605 plus sorafenib. Tumors were monitored twice weekly. As previously described [54], tumor size was measured on two axes with the aid of Vernier calipers and tumor volume (mm^3^) was calculated using the formula: 1/2(L × W^2^) where L is the longest and W is the shortest axis. Mice were euthanized at the end of the study and/or when tumor size exceeded 2,000 mm^3^. For tumor growth inhibition (TGI), the antitumor effects are calculated by dividing the tumor volumes from treatment groups by those of the control groups and multiplied by 100. The mice were examined frequently for overt signs of any adverse, drug-related side effects.

### TUNEL assay for apoptotic cells

The type of cell death (necrosis/apoptosis) was evaluated by the terminal deoxynucleotidyl transferase-mediated deoxyuridine biotin nick-end labeling (TUNEL) assay with an Apo-Direct kit (Pharmingen, San Diego, CA) as previously reported [55]. Briefly, after antigen retrieval, the tumor sections (4 μm-thick) were fixed by incubation with 4% paraformaldehyde at 4°C. The permeabilized sections were incubated with terminal deoxynucleotidyl transferase recombinant (rTdT) enzyme-catalysed reaction and nucleotide mixture for 60 min at 37°C in the dark. After immersion in stop/wash buffer for 15 min at room temperature, the sections were washed with PBS to remove unincorporated fluorescein-12-dUTP and the nuclei counterstained with hematoxylin.

### Immunohistochemical detection of Ki-67-positive cells

After fixation, tumor sections (4 μm thick) were deparaffinized and rehydrated, as described previously [55]. Following rehydration, antigen retrieval was carried out by placing the slides in 10 mmol/l sodium citrate buffer (pH 6.0) at 95°C for 20 min followed by 20-min cooling. The sections were then washed in PBS and non-specific binding sites were blocked with 1% bovine serum albumin with 2% goat serum in PBS before incubation with anti-Ki-67 antibody. After washing, the sections were incubated with biotinylated secondary antibody followed by horseradish peroxidase-conjugated streptavidin. The sections were further incubated with 2, 4-diaminobenzidine substrate and counterstained with hematoxylin.

### Statistical analysis

Data are presented as mean ± SD unless otherwise indicated. The statistical significance of the difference between the values of control and treatment groups was determined by either Student *t* test or simple one-way ANOVA followed by Tukey's *post hoc* test for multiple comparisons using Prism version 5 (GraphPad Software, Inc.). Values of *p*<0.05 were considered statistically significant.

## SUPPLEMENTARY MATERIAL FIGURES


