# Supplementary material for: Sorafenib and DE605, a novel c-Met inhibitor, synergistically suppress hepatocellular carcinoma
Source: Oncotarget. 2015 Mar 26;6(14):12340–56. doi: 10.18632/oncotarget.3656 (PMC4494942; doi:10.18632/oncotarget.3656)
Supplement: Supplementary file 1 [file oncotarget-06-12340-s001.pdf]

Sorafenib and DE605, a novel c-Met inhibitor, synergistically suppress hepatocellular carcinoma

Supplementary Material

Supplemental Figure S1

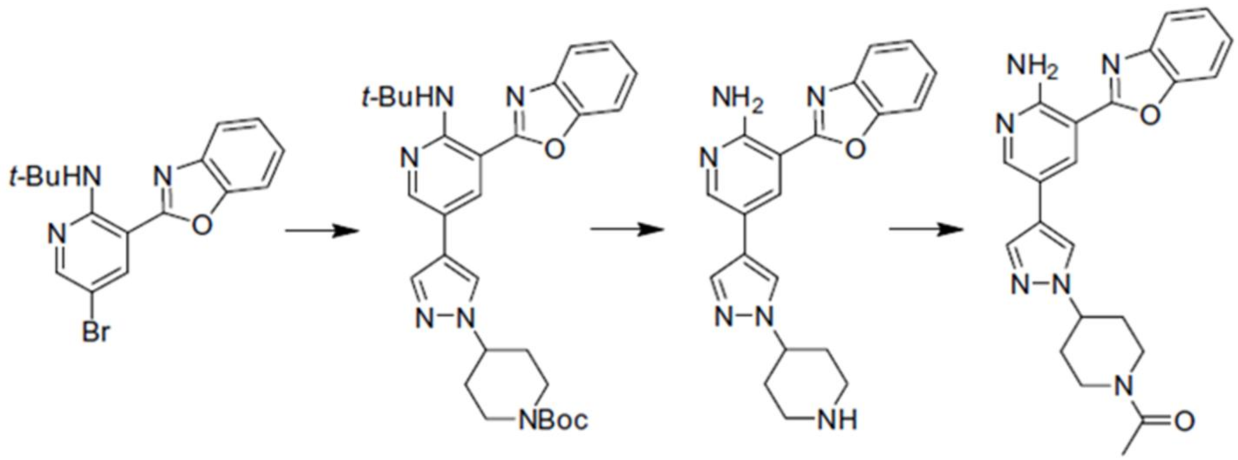

1 **2** **3** **DE605**

Supplemental Figure S1: Chemical structure and scheme of DE605 ((4-(4-(6-amino-5-(benzo[d]oxazol-2-yl)pyridin-3-yl)-1H-pyrazol-1-yl)piperidin-1-yl)ethanone) synthesis.

Supplemental Figure S2

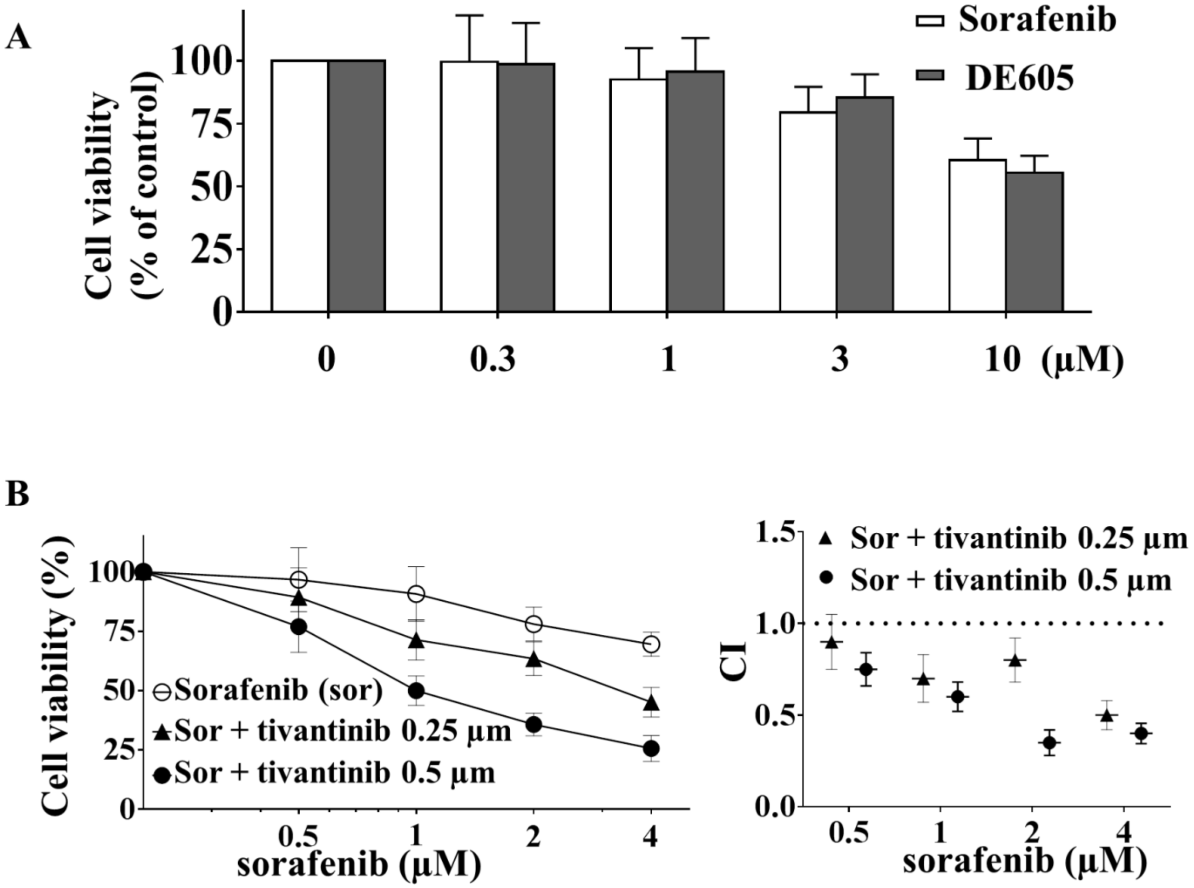

Supplemental Figure S2: Effects of DE605 on cell viability in normal liver cells and effects of sorafenib in combination with tivantinib on cell viability in hepatocellular carcinoma cells. (A) Normal liver cell line (HL-7702) were treated with different concentrations of DE605 or sorafenib for 72 hours, and cell viability was measured by MTT assay. (B) PLC/PRF/5 cells were treated with various concentrations of sorafenib in combination with tivantinib (ARQ 197) for 72 hours, and cell viability was measured by MTT assay (left). The combination index (CI) values were calculated by CompuSyn software (right). CI values <1 represent synergism, and the numbers reflect the corresponding data points (left). Each value represents the mean ± SD (n = 3).

Supplemental Figure S3

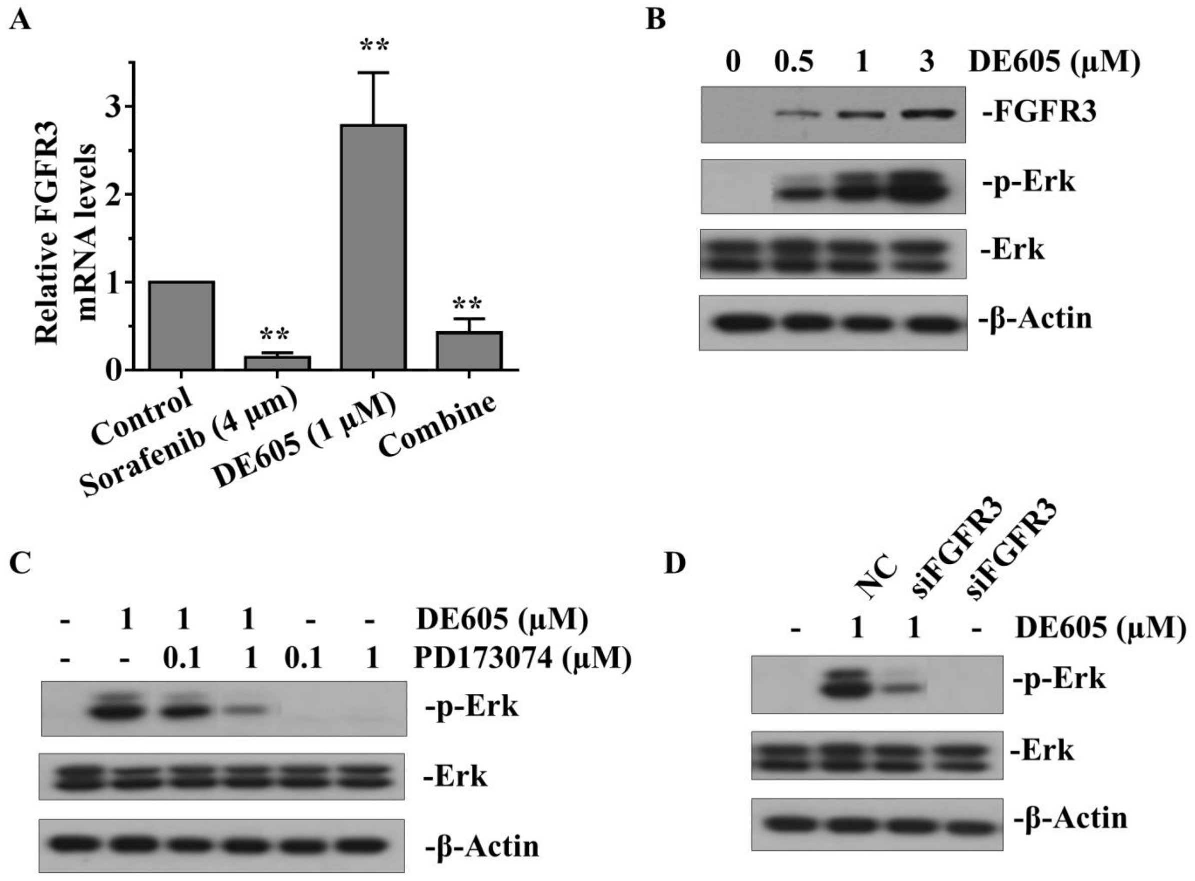

Supplemental Figure S3: Effects of sorafenib plus DE605 on signaling pathways in Hep3B cells. (A–D) Hep3B cells were treated with indicated agents for 72 hours or transfected with FGFR3 siRNA. Relative mRNA levels of FGFR3 were determined by RT-PCR (A). Data, mean ± SD (*n* = 3; **, *P*< 0.01 compared with the control group). Whole-cell lysates were subjected to western blotting analysis (B, C and D).
